# Flavonoids from Plants to Foods: From Green Extraction to Healthy Food Ingredient

**DOI:** 10.3390/molecules27092633

**Published:** 2022-04-20

**Authors:** Gerardi Carmela, Giovinazzo Giovanna

**Affiliations:** National Research Council-Institute of Science of Food Production (CNR-ISPA), Via Monteroni, 73100 Lecce, Italy

Research on flavonoids from plant sources is showing growing evidence of the versatile health benefits of flavonoids through in vitro and in vivo studies [1]. As the occurrence of flavonoids is directly associated with human daily dietary intake, it is important to evaluate flavonoid sources in food. However, there is still difficulty in accurately measuring the daily intake of flavonoids because of the complexity of the existence of flavonoids from various plant origins, and the occurrence of a large number of flavonoids itself in nature. Further achievements will undoubtedly lead to a deeper understanding the importance and stability [2] of flavonoids in either foods or pharmaceutical supplements.

This Special Issue harvested interesting advances in flavonoids with emphasis on green production and health aspects, which provide some suggestions for researchers and for industries in developing natural health agents. Research studies and reviews dealing with biological activity and healthy effects of polyphenols were also presented.

The first review of this Special Issue by Dias et al. [3] deals with the characterization of the most abundant natural flavonoids and their structure and chemical characteristics, extraction methods, and biological activity. In particular, this review reports on plants as a natural source of flavonoids as antioxidants. Flavonoids have the ability to control the accumulation of reactive oxygen species (ROS) via scavenger activity. Moreover, flavonoids are important secondary metabolites produced by plants with several functions related to the physiology of the growth and stress responses. This review presents the most abundant natural flavonoids (flavanols, flavonols, flavones, flavonones, isoflavons, anthocyanins); their structure and chemical characteristics; the plant, organ, and tissues of origin; the extraction methods; and biological activity. Vegetables, flowers, and seeds are rich in flavonoids, and methodologies to extract these compounds from these natural sources have been developed to be used for different purposes, such as food additives and preservatives. The awareness of the beneficial properties of flavonoids for human health (anti-inflammatory and anti-cancer actions, cardiovascular protection, antibacterial, antifungal, and antiviral activities) has triggered the increased consumption and interest in flavonoids uses in food processes and for therapeutic uses. However, the application of flavonoids for industrial purposes implies extraction processes with high purity and quality. Several methodologies have been developed aimed at increasing flavonoid extraction yield while still being environmentally friendly. The recognition of natural flavonoids as a good, safer source of antioxidants opens new perspectives to explore more of these compounds, focusing on new structures using new methodologies and technologies and exploiting other new natural sources.

A review by Giovinazzo et al. [4] aims to discuss the published background on the possible effectiveness of polyphenols to fight SARS-CoV-2 infection, contributing to the reduction in inflammation. Some of the anti-inflammatory therapies are discussed and although great progress has been made throughout the last year, there is no proven cytokine-blocking agents for COVID-19 currently used in clinical practice. The following were presented: potential anti-inflammatory compounds against SARS-CoV-2; polyphenols as natural molecules with anti-inflammatory activity; polyphenols and cytokines modulation; active anti-inflammatory plant extracts; and anti-inflammatory and viral activity of resveratrol. In this regard, bioactive phytochemicals such as polyphenols may become promising tools to be used as adjuvants in the treatment of SARS-CoV-2 infection. Such nutrients with anti-inflammatory and antioxidant properties and associated to classical anti-inflammatory drugs could help in reducing the inflammation in patients with COVID-19. No effective prophylactic or post-exposure treatments are available, although some anti-inflammatory compounds are currently in clinical trials. Studies of plant extracts and natural compounds show that polyphenols can play a beneficial role in the prevention and the progress of chronic diseases related to inflammation. Some of the anti-inflammatory therapies currently under investigation have been discussed, and novel therapies have been proposed. In particular, some IL-6 blocking agents are currently a matter of debate and more clinical trials are needed to better understand the feasibility of using such compounds in clinical practice. In this regard, bioactive phytochemicals, such as polyphenols may become promising tools for the treatment of COVID-19 in reducing the hyperactivation of cytokines, such as TNFα, IL-1β, IL-6, and IL-8. Such nutrients with anti-inflammatory and antioxidant properties may prevent or attenuate the inflammatory and vascular manifestations associated with COVID-19. Moreover, following healthy dietary patterns may have beneficial effects to contrast infection but still need to be explored.

Following the anti-inflammatory function of naturally occurring polyphenol molecules, the study of Zhang et al. [5] focused on the protection of the gastrointestinal mucosa against ulcer by natural flavonoids. A combination of flavonoids and existing drugs or the nanoencapsulation of flavonoids were found to exhibit better therapeutic effects on peptic ulcer when compared to the single or standard treatment. Flavonoids exhibit several anti-ulcer protective mechanisms, such as anti-acid secretory activity, cytoprotective effects, antioxidative activity, anti-inflammatory activity, and antibacterial activity. Flavonoids exert cytoprotective and rehabilitative effects by not only strengthening defense factors, such as mucus and prostaglandins, but also by protecting against potentially harmful factors via their antioxidative, anti-inflammatory, and antibacterial activities. Peptic ulcer disease is a common gastrointestinal tract disorder that affects up to 20% of the population of the world. The treatment of peptic ulcer remains challenging due to the limited effectiveness and severe side effects of the currently available drugs. Hence, natural compounds, owing to their medicinal, ecological, and other safe properties, are becoming popular potential candidates in preventing and treating peptic ulcers. Natural flavonoids, the most abundant polyphenols in plants, exhibit gastroprotective effects against peptic ulcer both in vivo and in vitro. In this review, the anti-ulcer functions and mechanisms, as well as their bioavailability, efficacy, and safety of flavonoid monomers in the gastrointestinal tract were summarized. The author listed a total of 60 kinds of flavonoids which exerted gastroprotective effects in different peptic ulcer models. In recent years, more reports that flavonoids also have remarkable therapeutic effects on peptic ulcer have gradually emerged, although they were more often proved in vitro, which showed that flavonoids have a prospect in treatments after injury. However, patients with peptic ulcer are underrepresented in clinic trials. At present, most data come from laboratory model tests. Moreover, the in vivo models of peptic ulcer include ulcers caused by oxidative damage, ethanol, NSAIDs, stress and Helicobacter pylori or acid-ethanol (ethanol or ethanol/HCl)-induced acute gastric ulcer models. These models reflected the causes and phenotypes of human peptic ulcer disease, although the protective effects of the flavonoids in these models are often determined by the route of administration, animal species, duration, and the dose of administration. Although future controlled clinical studies and bioavailability improvements are needed to assess the efficacy of flavonoids in preventing and/or treating peptic ulcer disease, it is still undeniable that flavonoids, especially the monomers, are suitable candidates in preventing as well as treating peptic ulcers.

The study of Gerardi et al. [6] investigates the antioxidant and antimicrobial activities of whole (skins and seeds) and separated skin pomaces from fermented (red Negramaro cv) and unfermented (white Fiano cv) grape by-products. The antioxidant activity, measured by both ORAC and TEAC assays, was higher in whole than in skin pomace extracts. The HPLC characterization of the phenolic composition in whole and skin pomace extracts confirms grape by-products as one of the main sources of flavonoids, namely, anthocyanins in red skin pomace and flavanols in white skin pomace. Red and white skin pomace extracts showed a Minimum Inhibitory Concentration (MIC) of 31.25–62.5 GAE/mL against Staphylococcus aureus and Enterococcus faecalis. Pseudomonas spp. were more sensitive to red skin pomace extracts rather than white skin pomace extracts. This article demonstrates that lyophilized grape pomace extracts could be used as natural preservatives in the food industry. Plant bioactive molecules can be produced by renewable and low-cost sources, and this plays an important value for the enhancement and the exploitation of by-products of food chains.

In the article of Piechocka et al. [7], the effect of the thiamine concentration (hydrochloride-TH and pyrophosphate-TP) on the chelating properties, reducing power, and free radical scavenging indices of catechins and caffeine was reported. The study demonstrate that high concentration of TH and TP can reduce the antioxidant indices of catechins (EGCG, EGC, ECG) and caffeine. However, samples containing TH or TP in amounts ranging from 0.1 to 0.4 mg/100 g showed an increase in these indices. The findings of this study suggest the maximum amount of 6 mg/100 g of thiamine to obtain the optimum quality of supplemented foods containing catechins and caffeine.

The article of Saeting et al. [8] deals with the anti-diabetic effects of a mungbean water extract (MWE). The use of nonorganic solvent in the extraction method is noteworthy as it is recognized as safe for human consumption and sustainable for the environment. The isomeric flavonoids vitexin and isovitexin were identified in MWE using HPLC and NMR. MWE inhibited α-amylase, α-glucosidase, and the formation of advanced glycation end-products (AGEs). The study of Saeting et al. [8] shows that MWE improved the insulin sensitivity of insulin-resistant HepG2 cells and suppressed the mRNA expression of genes involved in glucose metabolism.

The study of Kim et al. [9] evaluates the in vitro effect of Allium senescens L. (A.S.) extract on cell survival and IL-2-mediated inflammation in human T cell acute lymphocytic leukemia (T-ALL) Jurkat cells. The study demonstrated that A.S. extract induced caspase-dependent apoptosis and suppressed the expression and secretion of IL-2 mRNA via the inhibition of the NF-κB signaling pathway in T-ALL Jurkat cells. Moreover, a strong synergistic effect has been demonstrated between anticancer substances such as abitinib and A.S. extract. This study showed that A.S. extract might be used for the development of new natural-product-derived drugs.

In the research article by Shen T. et al. [10], a study on flower petals from four Chaenomeles species with gradual difference in color is presented with the aim to gain a better understanding of the flavonoid metabolism in flower petals. This study provides new insights into the development and utilization of Chaenomeles petals and provides a basis for future investigations into their utilization as a flavonoid source. The screening of differential flavonoid metabolites showed that Chaenomeles species with the highest pigmentation of flower petals had higher levels of flavonoid metabolites when compared with the other three Chaenomeles species. Annotation and enrichment analysis of flavonoid metabolites revealed that anthocyanins are likely responsible for the colour differences of the four Chaenomeles petals. Additionally, a large number of flavonoids, flavonols, and isoflavones were enriched in the petals of highest pigmented Chaenomeles. Annotation and enrichment analysis of flavonoid metabolites revealed that cyanidin 3,5-diglucoside and pelargonidin-3,5-diglucoside anthocyanins are likely responsible for the color differences of the four Chaenomeles petals, as well as a large number of flavonoids, flavonols, and isoflavones. This study provides new insights into the development and utilization of Chaenomeles petals and provides a basis for future investigations into their utilization.
